# Use of Nasal Non-Invasive Ventilation with a RAM Cannula in the Outpatient Home Setting

**DOI:** 10.2174/1874306401711010041

**Published:** 2017-07-21

**Authors:** Wilfredo De Jesus Rojas, Cheryl L. Samuels, Traci R. Gonzales, Katrina E. McBeth, Aravind Yadav, James M. Stark, Cindy Jon, Ricardo A. Mosquera

**Affiliations:** University of Texas Health Science Center, McGovern Medical School, Houston, Texas, USA

**Keywords:** Nasal Non-Invasive Ventilation, RAM Cannula, Chronic respiratory failure, Pediatric Complex Care, Tracheostomy Avoidance

## Abstract

**Background:**

Nasal non-invasive-ventilation (Nasal NIV) is a mode of ventilatory support providing positive pressure to patients via a nasal interface. The RAM Cannula is an oxygen delivery device that can be used as an alternative approach to deliver positive pressure. Together they have been successfully used to provide respiratory support in neonatal in-patient settings.

**Objective:**

To describe the outpatient use of Nasal NIV/RAM Cannula as a feasible alternative for home respiratory support in children with chronic respiratory failure.

**Methods:**

We performed a retrospective case series of 18 children (4 months to 19 years old) using the Nasal NIV/RAM Cannula in the Pediatric Pulmonary Clinic at the McGovern Medical School, UTHealth (2014-16). Consideration for Nasal NIV/RAM Cannula utilization included: inability to wean-off in-patient respiratory support, comfort for dyspnea, intolerability of conventional mask interfaces and tracheostomy avoidance.

**Results:**

Average age was 7 years. 50% were Caucasian, 38% African-American and 11% Hispanics. Pulmonary disorders included: chest wall weakness (38%), central control abnormalities (33%), obstructive lung disease (16%) and restrictive lung disease (11%). Indications for Nasal NIV/RAM Cannula initiation included: CPAP/BPAP masks intolerability (11%), dyspnea secondary to chest wall weakness (38%) and tracheostomy avoidance (50%). Average length of use of Nasal NIV/RAM Cannula was 8.4 months. Successful implementation of Nasal NIV/Ram Cannula was 94%. One patient required a tracheostomy following the use of Nasal NIV/RAM Cannula. Significant decrease in arterial PaCO_2_ pre and post Nasal NIV/RAM cannula initiation was notable (p=0.001).

**Conclusion:**

Outpatient use of Nasal NIV/RAM Cannula may prove to be a feasible and save treatment alternative for children with chronic respiratory failure, chest wall weakness, dyspnea and traditional nasal/face mask intolerance to avoid tracheostomy.

## INTRODUCTION

Nasal non-invasive ventilation (Nasal NIV) is a ventilatory support mode that provides positive pressure to patients via a nasal cannula or mask. Continuous positive pressure can be delivered using several treatment modalities, most commonly by Continuous Positive Airway Pressure (CPAP) or Bi-level Positive Airway Pressure (BPAP). NIV has been shown to be an effective means of providing respiratory support in the acute care setting to prevent endotracheal intubation [1]. More recently, the use of Nasal NIV has been associated with improved outcomes in the neonatal intensive care unit [2, 3]. Furthermore, Nasal NIV using a RAM Cannula [Neotech] interface (Nasal NIV/RAM Cannula) has been shown to decrease the need for endotracheal intubation in neonates after acute respiratory failure [4]. Depending of the underline pulmonary pathophysiology of the disease, patients may require a long-term respiratory support. Available options to provide outpatient minimal positive pressure support in children is limited. Nasal NIV/ RAM Cannula may be a feasible and save long-term respiratory support for outpatient.

The concept of RAM Cannula was first introduced as a Nasal NIV device in 2011 for infants in the neonatal intensive care unit. Although initially created for oxygen delivery, ex vivo experiments and published data with neonates intensive unit demonstrated that RAM Cannula has the potential to improve patient tidal volume, decrease work of breathing and improve ventilation and oxygenation facilitating extubation [5, 6]. When compared to the traditional nasal cannula, the RAM Cannula is made of a more flexible, softer material with thinner walled nasal prongs. Due to the increased diameter of the inner nasal prongs in the RAM Cannula, a decrease in airflow resistance is allowed when compared to a traditional nasal cannula. Studies evaluating the delivery of positive pressure using a RAM Cannula in a lung simulator have shown that with proper fitting of prongs in relation to the patient nasal diameter, around 60-70% of the positive pressure can be transmitted across the interface [5]. The opportunity to provide positive pressure ventilation with the RAM Cannula may be a useful characteristic for patient with chronic respiratory diseases requiring long term respiratory support.

Depending on the underlying pulmonary pathophysiology, patients may require positive pressure for long-term management. At this time, the availability of Non-invasive ventilation (NIV) interfaces that can be used in the outpatient setting is limited to BPAP or CPAP with either a traditional nasal or full-face mask. These approaches can restrict the use of positive pressure delivery devices in the pediatric outpatient setting due to several factors including: patient age, weight, proper fit of mask, patient intolerance and comfort with the interface, facial irritation, mid-face hypoplasia and skin breakdowns [7, 8]. RAM cannula may be an alternative interface to address most current pediatric concerns with traditional interfaces.

Some patients require minimal positive pressure for respiratory support secondary to obstructive and restrictive lung disease, chest wall weakness or due to a poor central control respiratory drive. Those patients with chronic respiratory failure may benefit from the use of Nasal NIV/RAM Cannula in the outpatient setting as an alternative for respiratory support. Our goal is to describe and discuss the feasibility of Nasal NIV/RAM Cannula in children with chronic respiratory failure.

## MATERIALS AND METHODS

A retrospective case series of 18 medical records were reviewed to identify patients on Nasal NIV/RAM Cannula [Neotech] at the outpatient pediatric pulmonary and High Risk Children clinics of the McGovern Medical School at UTHealth located in Houston, Texas. The UTHealth Institutional Review Boards approved the data collection and analysis for the Protection of Human Subjects. Collected information included patient demographics, clinical diagnoses, Nasal NIV/RAM Cannula mode of ventilation with parameters, blood carbon dioxide levels (PaCO_2_), oxygen saturation (SO_2_), associated complications and related side effects. Data was obtained from the electronic medical records for a time frame of two years (2014-2016). Pediatric patients between 0 to 21 years old on Nasal NIV/RAM Cannula were included. Patient using other interfaces for respiratory support rather than RAM Cannula were excluded.

### Nasal NIV/RAM Cannula Patient Considerations

Indications to initiate Nasal NIV/RAM Cannula in our patients were based on the underlying respiratory lung disease that included chest wall weakness, central control abnormalities, obstructive lung disease and restrictive lung disease. Other considerations were the amount of positive pressure required on previous NIV, the inability to wean off from NIV respiratory support, including CPAP, BPAP or High flow nasal cannula (HFNC) in the inpatient setting, a history of chronic hypoventilation, as an option to prevent tracheostomy and as a comfort measure in patients with dyspnea secondary to the underlying respiratory disorder. The PaCO_2_ data was collected before and after Nasal NIV/RAM cannula initiation.

### Data Analysis

Descriptive statistics were obtained and presented in percentages and means. Comparison of PaCO_2_ data between groups was analyzed using a paired t-test. AP-value of less than 0.05 was considered to be statistically significant.

## RESULTS

### Patient Characteristics

A total of 18 pediatric patients with a mean age of 7 years met inclusion criteria for the study. A total of 61% of our cohort was female and patients were using Nasal NIV/RAM Cannula for a total average of 8.4 months. All patients used a Nasal NIV/RAM Cannula with a Trilogy portable mechanical ventilator [Philips Respironics]. The most common NIV modes utilized were: BPAP (66%) and CPAP (23%) followed by Synchronized Intermittent Mandatory Ventilation – Pressure Control (SIMV-PC) (11%). Average settings for BPAP were Inspiratory Positive Airway Pressure (IPAP): 15 cmH_2_O, Expiratory Positive Airway Pressure (EPAP): 6 cmH2O and a Respiratory Rate (RR) of 13 breaths per minute. When using the CPAP mode, the average setting was 7 cmH_2_O. The underline respiratory lung disease varies among patient using CPAP or BPAP mode. Analysis of PCO_2_ levels showed a significantly lower PCO_2_ levels after Nasal NIV/RAM Cannula initiation (p=0.001) in our cohort. Average SO_2_ of patients at room air was 98% on Nasal NIV/RAM Cannula. In our study population, 38% of subjects reported seizure episodes and 50% experienced hypoxemia prior to initiation of Nasal NIV/RAM Cannula.

Baseline characteristics and a summary of the data collected for our cohort are presented in Table **1**. Baseline respiratory lung disease in our sample included the following: chest wall weakness (38%), respiratory central control disorders (33%), obstructive lung disease (16%) and restrictive lung disease (11%).

In our cohort, 22% of the patients had dyspnea associated with underlying mitochondrial disorders. Respiratory muscle strength studies were completed on a total of four patients. All of them had a lower percent predicted for Maximum Expiratory and Inspiratory Pressures (MEP/MIP). Two patients with mitochondrial disorders were on full mechanical ventilation through Nasal NIV/RAM Cannula using SIMV-PC, RR: 22/min, PC: 22 cmH2O, PEEP: 0 cmH2O, Inspiratory time (IT): 0.7 seconds, Fraction of Inspiratory Oxygen (FIO2): 1-2 Liters per minute.

Obstructive disorders in our cohort included tracheomalacia or laryngomalacia (11%) and obstructive sleep apnea (OSA) (38%). We included a 4 month-old male with Trisomy 21, failure to thrive, and severe laryngomalacia status post supraglottoplasty. Prior to initiation of Nasal NIV/RAM Cannula, the patient was unable to gain weight and there were considerations for tracheostomy. On Nasal NIV/RAM Cannula, weight gain was documented and avoided tracheostomy placement.

Central control disorders also increase the risk of hypoventilation leading to hypercapnea and hypoxemia. Thirty three percent of our patients have central control disorders such as ataxia, seizures, cerebral palsy, dysautonomia, anoxic brain injury or hypoxic ischemic encephalopathy. In these patients, avoidance of tracheostomy was an important consideration in choosing how to manage their long-term respiratory support.

Additionally, two patients with restrictive lung disease secondary to scoliosis also had progressive neuromuscular weakness of unknown origin where able to improve dyspnea symptoms using Nasal NIV/RAM Cannula.

### Nasal NIV/RAM Cannula Indication

Analysis of the initial consideration to initiate Nasal NIV/RAM Cannula in chronic respiratory patients included: avoidance of tracheostomy (for medical reasons including poor prognosis or parental refusal) (50%), to decrease dyspnea and improve comfort secondary to chest wall weakness (38%) and patient intolerance to conventional respiratory support modes (CPAP/BPAP) or interfaces (11%).

### Complications and Side Effects Reported

Complications associated with Nasal NIV/RAM Cannula were negligible in our study population. A minimal nasal rub on the nasal columella was reported as an adverse side effect in one patient. Most of the patients’ caretakers endorsed good adherence and adequate patient tolerance with Nasal NIV/RAM Cannula.

## DISCUSSION

NIV is an essential tool in providing positive pressure ventilation to children with chronic respiratory failure in the home setting. Limited literature has been published about the use of RAM cannula as a NIV alternative. This study explores the incorporation of new technologies to provide respiratory support and introduces the idea of potential applications of Nasal NIV/RAM Cannula in a selected group of pediatric patients who have complex respiratory lung diseases. As previously described, the RAM Cannula has successfully been used to reduce the number of extubation failures in the neonatal intensive care(2). Patients with chronic respiratory failure requiring long term positive pressure ventilation, may also be able take advantage of Nasal NIV/RAM Cannula as a home respiratory support device.

Currently, options for pediatric patients who require outpatient long-term mechanical ventilation with NIV are limited to nasal or full-face mask. The most common cause of pediatric non-compliance with NIV is intolerability of facial or nasal mask. Due to intolerability, patients have to resort to invasive interventions that include a tracheostomy as the next step. It is known that tracheitis, tracheal decannulation and airway granulomas are some of the disadvantages of a long-term tracheostomy and add additional medical complications and morbidity to the child’s long-term care.

Nasal NIV/RAM Cannula may also play a role in the outpatient weaning of respiratory support initiated in the hospital. Some patients require minimal support (HFNC on minimal support, CPAP or BPAP). Unfortunately hospital devices such as HFNC are only approved in the inpatient setting. Due to patient safety concerns, and lack of portability, devices such as HFNC are not viable options for long-term respiratory support in children. Additionally, the long-term use of conventional nasal and full-face mask in pediatric patients has been associated with an increased risk of aspiration, impairment of facial bone development, skin breakdown and infections [7, 8]. We found Nasal NIV/RAM Cannula to be an effective device decreasing PCO_2_ in 18 complex care children with chronic respiratory failure from age 4 months to 19 years old. Nasal NIV/RAM Cannula provided a gentle interface that is capable of delivering positive pressure (BPAP, CPAP, SMIV-PC) and decrease hypercapnea with minimal complications in the home setting.

We understand that comfort at the end of life is important for both the patient and the family. Avoidance of traditional CPAP/BPAP interfaces that can potentially create skin abrasions, facial pressure ulcers, and perceived discomfort may help decrease the stress associated with a chronic illness. Nasal NIV/RAM Cannula is a potential option to avoid seemingly uncomfortable NIV or tracheostomy and its associated complications. Nasal NIV/RAM Cannula could possibly serve as an alternative home respiratory support in patients who are receiving compassionate end of life care.

Nasal NIV/RAM Cannula may be an additional consideration in the algorithm before considering tracheostomy in medically complex children. Although our cohort was limited to 18 patients, our experience validates the feasibility of Nasal NIV/RAM Cannula as an alternative to conventional forms of NIV, which may be poorly tolerated. Additionally, avoidance of tracheal cannula placement is ideal, as it would prevent the additional morbidity, mortality, and long-term consequences associated with tracheostomies. This study explored and found minimal side effects with no significant major complications or mortality with Nasal NIV/RAM Cannula over a period of two years. Additionally, patients demonstrated good tolerability of the device as reported by caretakers. We found Nasal NIV/RAM Cannula to be a viable option to consider when managing long-term positive pressure ventilation in children with complex pulmonary disease.

Our study has some limitations. The power of the sample is small secondary to the quantity of patients currently using this specific technology. A larger study with additional subjects is needed to confirm our findings. Also, the average length of use of the Nasal NIV/RAM Cannula in our cohort was limited to 8.4 months. It is possible that long-term complications and potential side effects may arise after long-term use.

## CONCLUSION

Use of Nasal NIV/RAM Cannula in the outpatient setting may prove to be beneficial for patients with chronic respiratory failure. Further studies are required to evaluate long-term outcomes, complications, morbidity and mortality in medically complex, technology dependent children.

## Figures and Tables

**Table 1:** Subjects clinical characteristics.

*Clinical characteristics*		*Value (N=18)*
Gender (F/M), N (%)		11(61%) / 7(38%)
Age, mean ± SD, years		7 ± 6.3
Ethnicity, N (%)		
Caucasian		9(50)
African-American		7(38)
Hispanics		2(11)
Nasal NIV RAM Cannula use;		
mean ± SD, months		8.4 ± 7.3
Pathophysiologic Disorder (%)		
Obstructive Lung Disease		3(16)
Restrictive Lung Disease		2(11)
Central Control Disease		6(33)
Chest Wall Weakness		7(38)
Nasal NIV/RAM Cannula Trilogy Mode, N (%)		
BPAP		12(66)
CPAP		4(23)
SIMV-PC		2(11)
Arterial Carbon Dioxide tension (PaCO_2_) *		
mean ± SD, mmHg		
Before Nasal NIV/RAM Cannula		56.1 ± 12.8
After Nasal NIV/RAM Cannula		43.2 ± 8.3
Percent of saturation on Nasal NIV/RAM Cannula		
mean ± SD, percent (%)		98 ± 1.4
MEP/MIP, mean ± SD, N=4		
MEP (cmH_2_O)		32.7 ± 8.4
MIP (cmH_2_O)		55.2 ± 20.5
Indications for Nasal NIV/RAM Cannula Use, N (%)		
Avoidance of tracheostomy		9(50)
Dyspnea comfort		7(38)
Intolerability to conventional support		2(11)

F, Female; M, Male; SD, standard deviation; Nasal NIV/RAM Cannula, Nasal Non Invasive Ventilation using RAM Cannula; BPAP, Bi-level Positive Airway Pressure; CPAP, Continuous Positive Airway Pressure; SIMV-PC, Synchronized Intermittent Mandatory Ventilation - Pressure-Control; MEP/MIP, Maximum Expiratory and Inspiratory Pressures. * (p=0.001).
